# Whiplash trauma did not predict jaw pain after 2 years: an explorative study

**DOI:** 10.1007/s00784-024-05555-z

**Published:** 2024-02-22

**Authors:** Alicia Böthun, Anna Lövgren, Britt-Marie Stålnacke, Ewa Lampa, Catharina Österlund, Birgitta Häggman-Henrikson, Fredrik Hellström

**Affiliations:** 1 Department of Odontology, Clinical Oral Physiology, Faculty of Medicine, Umeå University, 901 87 Umeå, Sweden; 2 Department of Community Medicine and Rehabilitation, Rehabilitation Medicine, Umeå University, Umeå, Sweden; 3 Department of Orofacial Pain and Jaw Function, Faculty of Odontology, Malmö University, Malmö, Sweden; 4 Department of Occupational Health Science and Psychology, Faculty of Health and Occupational Studies, University of Gävle, Gävle, Sweden

**Keywords:** Temporomandibular disorders, Whiplash injuries, Facial pain, Jaw pain, Neck pain

## Abstract

**Objectives:**

To explore predictive factors for the development and maintenance of jaw pain over a 2-year period.

**Methods:**

One hundred nineteen cases (73 women) and 104 controls (59 women), mean age 34.9 years (SD 13.9), attended baseline and 2-year follow-up examinations. The whiplash cases visited the emergency department at Umeå University Hospital, Sweden, with neck pain within 72 h following a car accident, and baseline questionnaires were answered within a month after trauma. Controls were recruited via advertising. Inclusion criteria were age 18–70 years, living in Umeå municipality and Swedish speaking. The exclusion criterion was neck fracture for cases and a previous neck trauma for controls. Validated questionnaires recommended in the standardized Research Diagnostic Criteria for temporomandibular disorders were used. Jaw pain was assessed by two validated screening questions answered with “yes” or “no.” A logistic regression analysis was used to predict the outcome variable jaw pain (yes/no) after 2 years.

**Results:**

Whiplash trauma did not increase the odds of development of jaw pain over a 2-year period (OR 1.97, 95% CI 0.53–7.38). However, non-specific physical symptoms (OR 8.56, 95% CI 1.08–67.67) and female gender (OR 4.89, 95% CI 1.09–22.02) did increase the odds for jaw pain after 2 years.

**Conclusion:**

The development and maintenance of jaw pain after whiplash trauma are primarily not related to the trauma itself, but more associated with physical symptoms.

**Clinical relevance:**

The development of jaw pain in connection with a whiplash trauma needs to be seen in a biopsychosocial perspective, and early assessment is recommended.

**Supplementary Information:**

The online version contains supplementary material available at 10.1007/s00784-024-05555-z.

## Introduction

Temporomandibular disorders (TMD) are the most common chronic orofacial pain condition where frequent symptoms are pain and dysfunction in the jaw muscles, the temporomandibular joint, or both [1]. The prevalence of TMD in the general population is estimated at 10%, with the highest prevalence among women and during working age [2, 3]. Approximately 20% of individuals with TMD onset will develop chronic jaw pain, and women have a higher risk of developing chronic jaw pain [4, 5]. However, currently, knowledge is sparse on risk factors for the development of chronic jaw pain.

TMD is considered to have a multifactorial etiology [6] and psychosocial risk factors such as depression and stress have been suggested [7]. Moreover, depression together with non-specific physical symptoms such as upset stomach, back pain, and perceived heaviness in extremities can affect the clinical assessment [8]. In line with this, non-specific physical symptoms and psychological factors have been identified as some of the more robust risk factors for the first onset of TMD [9]. Assessing non-specific physical symptoms and depression is part of standardized and internationally adopted protocols for TMD examinations [1, 10]. Furthermore, macro traumas, such as a whiplash trauma, are also suggested to be an aggravating factor to TMD [11] with a reported median prevalence of TMD after whiplash trauma of approximately 20% [12].

The term whiplash-associated disorders (WAD) describes a group of symptoms following whiplash trauma that is often attributed to a motor vehicle accident [13]. The most frequent WAD symptoms are neck pain, neck disability, and headache [14, 15]; however, pain can also occur in other body regions including the orofacial area [16]. The incidence of whiplash trauma is equal between genders [13], but women have a poorer prognosis for recovery [17] and are at a higher risk for developing chronic WAD symptoms [18]. The most commonly described risk factors for chronic symptoms following whiplash trauma, in addition to female gender, are high neck pain intensity and disability and severity of acute symptoms [18].

Most previous studies on the relationship between TMD and WAD have evaluated these conditions separately or in cross-sectional settings. It was suggested that TMD pain appears in close connection with a whiplash trauma [19, 20], but there is a lack of prospective studies evaluating the development of jaw pain after whiplash trauma. To provide a more comprehensive understanding of relevant factors, the aim of the present study was to explore predictive factors, including whiplash trauma, for the development and maintenance of jaw pain over a 2-year period.

## Methods

### Study population

**Fig. 1:**
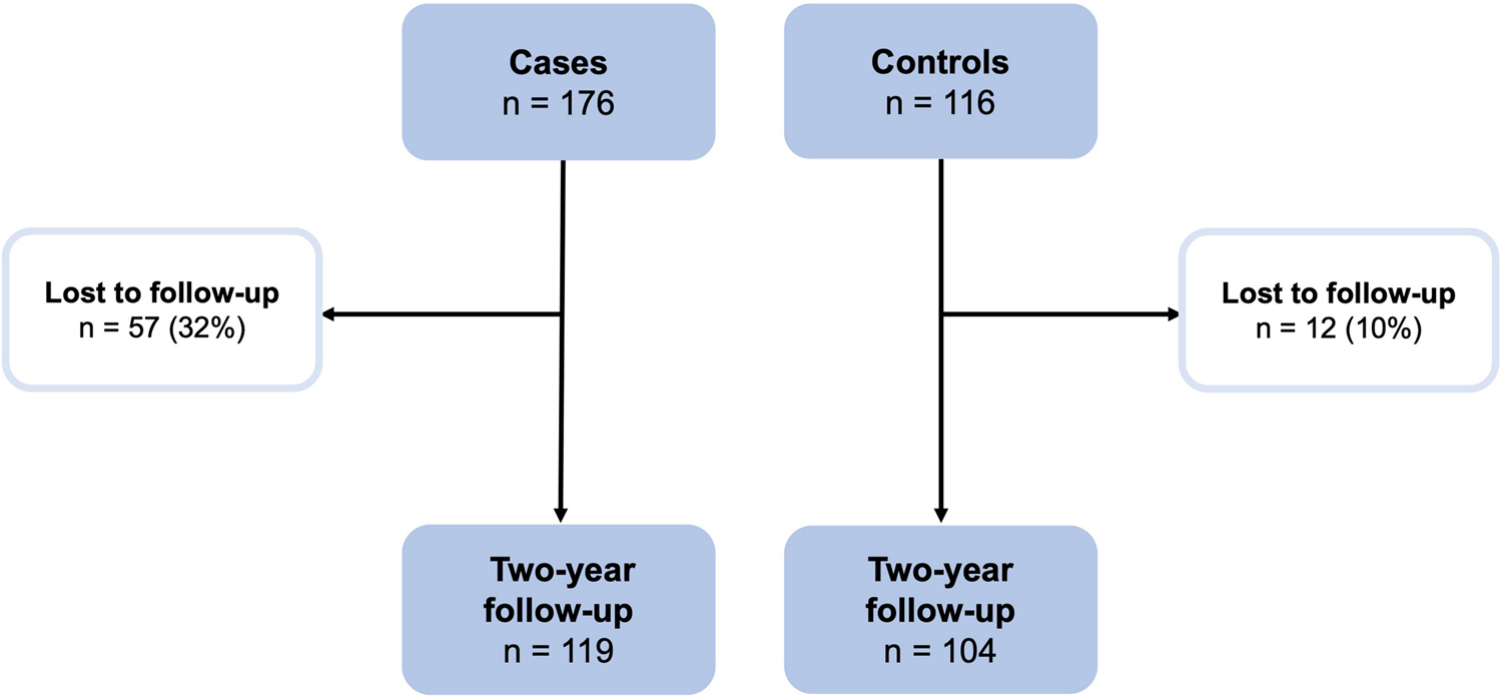
Flowchart of the cases and controls at baseline and at the 2-year follow-up

At baseline, there were 292 eligible individuals (176 cases, mean age 35.2 years SD 14.4; and 116 controls mean age 32.8 years SD 12.8). Of these, a cohort of 223 individuals (132 women and 91 men, mean age 36.9 years SD 13.9) that entailed 119 cases (73 women and 46 men, mean age 38.8 years SD 14.7) and 104 controls (59 women and 45 men, mean age 34.9 SD 14.7) attended the 2-year follow-up and were thus included in the analyses (Fig. 1). The cases had visited the emergency department at Umeå University Hospital, Sweden, with a whiplash trauma within 72 h following a car accident. Umeå is a mid-sized Swedish city located approximately 400 km south of the Arctic Circle. The city has only one hospital within a well-defined catchment area. The cases were prospectively recruited through the hospital’s Injury Data Base, and the controls were recruited from the general population via advertising.

The baseline assessment for cases was performed within a month after trauma between December 2010 and January 2016 (controls were recruited parallel to this time frame) and the 2-year follow-up was between December 2012 and January 2018. Inclusion criteria for both cases and controls were age 18–70 years, living in Umeå municipality and having an understanding of the written and spoken Swedish language. The exclusion criterion was a neck fracture (WAD grade IV) for cases and a previous neck trauma for controls. Participants received oral and written information and signed an informed consent prior to enrolment. The administration of invitations and questionnaires was managed by a dental nurse. During the data collection, group allocation (case/control) was blinded, and during analysis, the participants remained pseudoanonymous. The study was approved by the Regional Ethical Review Board in Umeå, Sweden (Dnr 2010–156-31 M), and was conducted in accordance with the World Medical Association Declaration of Helsinki and conformed to the Strengthening the Reporting of Observational Studies in Epidemiology (STROBE) guidelines [21].

### Patient-reported outcomes

Validated questionnaires were used that are recommended in the standardized examination Research Diagnostic Criteria for TMD (RDC/TMD) [1]. Depression and non-specific physical symptoms were assessed by the Symptoms Checklist-90-Revisited (SCL-90-R) [22] modified for the RDC/TMD [1]. The SCL-90-R is a 90-item checklist that regards how affected an individual is by specific symptoms in the last month [22]. Thirty-two questions from the SCL-90-R are included in the RDC/TMD that screen for depression (20 items) and non-specific physical symptoms (12 items). The subscale for non-specific physical symptoms consists of seven non-painful items; faintness, trouble getting breath, hot and cold spells, numbness and tingling in body parts, lump in throat, weakness in body parts, and heaviness in arms and legs. The subscale also contains five painful items: headache, chest pain, lower back pain, upset stomach, and sore muscles. A total score for all 12 items were included in the analyses and not individual items. In the present study, non-specific physical symptoms hereafter will be referred to as physical symptoms. The severity of each item rates from 0 (not at all) to 4 (very much), and normative data and cutoff scores have been provided [1]. The physical symptom scale has good validity and good to acceptable internal consistency [8]. The mean values are classified as “normal,” “moderate,” and “severe” for depression as < 0.535, > 0.535 < 1.105, ≥ 1.105, respectively, and for physical symptoms as < 0.500, > 0.500 < 1.000, and ≥ 1.000, respectively [1]. No data were assessed on the distribution of pain sites. In addition to the RDC/TMD questionnaires, the neck disability index (NDI) [23] and current neck pain intensity were assessed using the numeric rating scale (NRS). Cutoff scores in relation to interference with function are mild (NRS ≤ 5), moderate (6–7), and severe pain (≥ 8) [24].

### Outcome measure

Jaw pain was assessed by two questions on pain, answered with “yes” or “no,” from the screening questions for TMD (3Q/TMD) which are validated for TMD pain diagnosis [3, 25].

- Q1: Do you have pain in your temple, face, jaw, or jaw joint once a week or more?
- Q2: Do you have pain once a week or more when you open your mouth or chew?

Jaw pain was categorized as positive when either of the two questions was answered affirmatively.

### Statistics

Descriptive statistics were used to characterize the study sample and were presented as the means and SDs or medians and interquartile ranges when appropriate. A logistic regression model presented as odds ratios (OR) together with a 95% confidence interval was used to predict the dependent variable jaw pain (yes/no) at 2 years. Factors included in the analyses were group (case vs. control), gender (women vs. men), neck disability (NDI, 0–100), current neck pain intensity (NRS, 0–10), depression (SCL-90-R, 0–4), and physical symptoms (SCL-90-R, 0–4). The model was adjusted for age (years) and education level (elementary school/secondary school vs. university degree). Because of the non-linear relationship between age and jaw pain [3], age was modelled using restricted cubic splines with three nodes. The logistic regression model was statistically significant, *χ*^2^(13) = 73.23, *p* < 0.001.

Interaction terms between neck disability and group, neck disability and gender, physical symptoms and group, and physical symptoms and gender were included in the analyses. Individuals with missing data at follow-up were not included in the analyses. Before analysis, the plan for analysis was registered on osf.io (10.17605/OSF.IO/E2MKQ). Statistical analyses were performed using SPSS Statistics version 28.0.1.0 and R version 4.1.3. Figures were constructed in Prism Graph Pad version 9.1.1 and Microsoft PowerPoint version 16.75. For all tests, a *p*-value < 0.05 was considered statistically significant.

## Results

For the cohort (*n* = 223, 119 cases, *n* = 104 controls) at baseline, the median neck disability score (NDI, 0–100) was 4 (IQR 16), and median neck pain intensity (NRS, 0–10) was 0 (IQR 2). Among these, the range for a neck disability was 0–74, and for neck pain intensity 0–9. The median, interquartile range (IQR), and range separated for cases and controls at baseline and at follow-up are presented in Table 1.

**Table 1:** Median, interquartile range (IQR), and range for neck disability using the neck disability index (NDI, 0–100) and current neck pain intensity using the numeric rating scale (NRS, 0–10). Physical symptoms and degree of depression at baseline and at the 2-year follow-up. Physical symptoms and degree of depression at baseline and at the 2-year follow-up

	Cases (*n* = 119)		Controls (*n* = 104)	
	Baseline	Follow-up	Baseline	Follow-up
Neck disability (NDI)				
Median (IQR)	14 (22)	8 (22)	2 (4)	2 (8)
Range	0–74	0–72	0–30	0–24
Neck pain intensity (NRS)				
Median (IQR)	2 (3)	1 (2)	0 (0)	0 (1)
Range	0–9	0–8	0–6	0–3
Physical symptoms				
Normal *n* (%)	35 (29.4%)	45 (37.8%)	80 (76.9%)	70 (67.3%)
Moderate *n* (%)	49 (41.1%)	28 (23.5%)	14 (13.4%)	21 (20.2%)
Severe *n* (%)	35 (29.4%)	46 (38.6%)	10 (9.6%)	13 (12.5%)
Depression				
Normal *n* (%)	59 (49.6%)	52 (43.7%)	75 (72.1%)	66 (63.5%)
Moderate *n* (%)	28 (23.5%)	37 (31.1%)	21 (20.2%)	23 (22.1%)
Severe *n* (%)	32 (26.8%)	30 (25.2%)	8 (7.7%)	15 (14.4%)

**Fig. 2:**
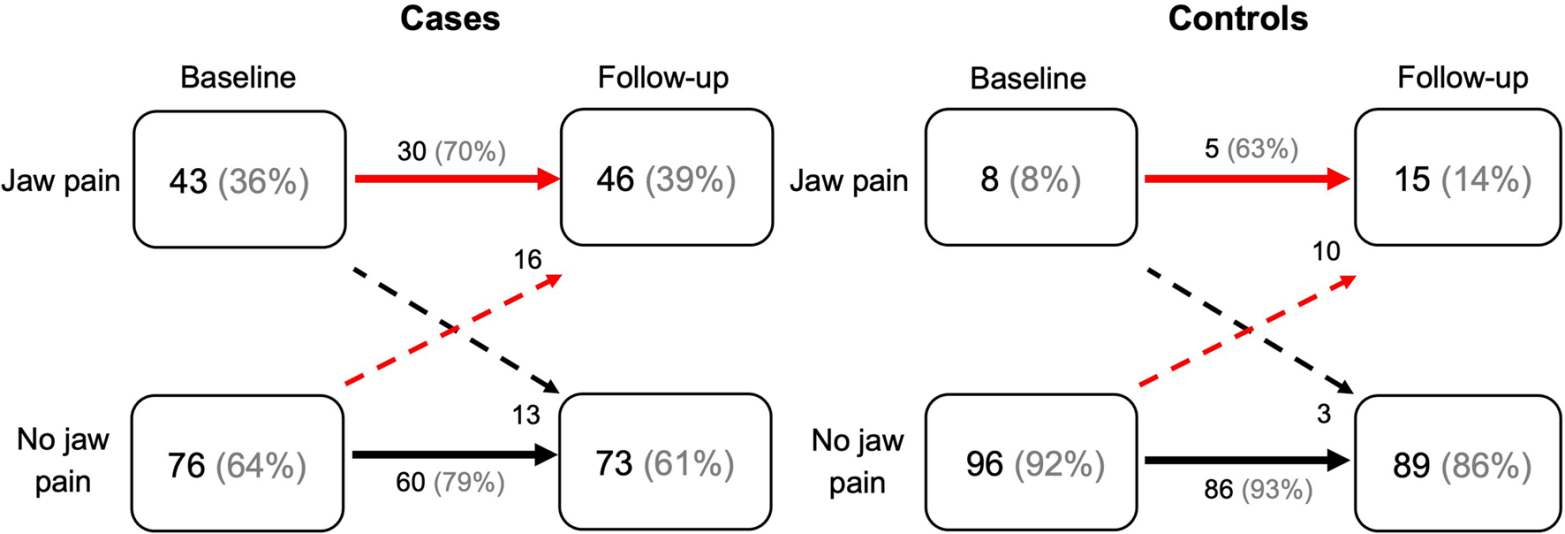
Flowchart of the outcome, jaw pain for cases (*n* = 119) and controls (*n* = 104) at baseline and at the 2-year follow-up

At baseline, 28.3% of the individuals in the cohort (*n* = 223) had moderate physical symptoms, and 20.2% had severe physical symptoms. For degrees of depression, 22.0% of the individuals in the cohort had a moderate degree, and 17.9% had a severe degree. The distribution of physical symptoms and degree of depression among cases and controls at baseline and at 2-year follow-up is presented in Table 1. At baseline, 36% of cases and 8% of controls reported jaw pain. Of these, approximately 70% of cases and 60% of controls also reported jaw pain at the 2-year follow-up (Fig. 2).

A higher score for physical symptoms (OR 8.56 95% CI 1.08–67.67) and female gender (OR 4.89, 95% CI 1.09–22.02) increased the odds of jaw pain after 2 years (Table 2). The cases, i.e., individuals with whiplash trauma, did not have increased odds for jaw pain 2 years after the trauma (OR 1.97, 95% CI 0.53–7.38) (Table 2).

**Table 2:** Logistic regression analysis for predicting factors for jaw pain. The model was adjusted for age and education level. The bold text indicates significance at *p* < 0.05

Predictors	Odds ratios	95% confidence intervals
Intercept	0.28	0.02–4.80
Group (whiplash)	1.97	0.53–7.38
**Gender (female)**	**4.89**	**1.09–22.02**
Depression	1.04	0.49–2.19
**Physical symptoms**	**8.56**	**1.08–67.67**
Current neck pain intensity	0.88	0.59–1.32
Neck disability	1.09	0.95–1.25
Group (whiplash)*physical symptoms	0.44	0.07–2.80
Gender (female)*physical symptoms	0.57	0.12–2.73
Group (whiplash)*neck disability	1.01	0.89–1.15
Gender (female)*neck disability	0.96	0.88–1.04

The interaction between gender and physical symptoms did not show increased odds for jaw pain after 2 years (OR 0.57, 95% CI 0.12–2.73) and neither did the interactions between group and physical symptoms, group and neck disability, and gender and neck disability (Table 2). Age and education level were not associated with an increased likelihood of jaw pain after 2 years. The logistic regression model explained 40.5% (Nagelkerke *R*^2^) of the total variance in the data.

## Discussion

From this explorative study that evaluated predictive factors for the development and maintenance of jaw pain, the main finding was that a previous whiplash trauma alone did not predict jaw pain over a 2-year period. Physical symptoms and female gender did however increase the risk for jaw pain, regardless of the other evaluated factors.

### Whiplash trauma

A higher frequency of jaw pain after whiplash trauma has been reported, predominately based on studies on individuals with chronic WAD (12). In the present study, cases had a higher prevalence of jaw pain (36%) than controls (8%) already in the acute stage 1 month after the trauma (baseline) and more than two-thirds of these cases (70%) reported jaw pain also at the 2-year follow-up. Having experienced a whiplash trauma 1 month earlier did not however predict further development of jaw pain from the 1-month post-trauma baseline over a 2-year period. Furthermore, in a subsample of this study population, there was no time effect seen for pain on palpation of the jaw or neck muscles between the 1-month baseline after the whiplash trauma and the 2-year follow-up [26]. Taken together, this suggests that jaw pain and disability develop in the early acute stage after whiplash trauma and can be detected already within a month of trauma. This finding, together with the fact that no substantial improvement with regard to jaw pain was seen over a 2-year period, strongly suggests an early assessment after whiplash trauma should include dentists specialized in orofacial pain.

Although an association between WAD and TMD has been proposed [11, 19], a causal relationship has not been demonstrated. Support of an association between WAD and TMD is founded in the close neurophysiologic associations between the jaw and neck regions, where trigeminal and cervical afferents converge in the caudal portion of the trigeminal brainstem sensory nuclei and on upper cervical nociceptive neurons [27, 28]. This neurophysiological relationship provides a foundation for the possible spread of pain between the jaw and neck regions [27]. Jaw pain among patients with chronic WAD has also been suggested to be part of a more widespread pain condition [29]. Taken together with the higher psychological distress among chronic whiplash patients [29], other factors such as gender [30] and physical symptoms [9] are possibly more important for pain development and maintenance in the jaw region than the whiplash trauma itself.

### Physical symptoms

A high level of physical symptoms as a predictor for the development of jaw pain is in line with previous studies where both psychological distress and physical symptoms were reported as risk factors for jaw pain [31, 32]. In addition, individuals with TMD show higher levels of physical symptoms when compared to controls [33]. Physical symptoms were also a risk factor for incident TMD regardless of whether pain was included as a physical symptom or not [9]. In a systematic review, the prevalence of moderate-severe physical symptoms ranged from 28.5 to 76.6% among TMD patients [34]. Physical symptoms thus seem to go hand in hand with changes in TMD, i.e., if one increases so does the other [31]. In our study, the prevalence of moderate-severe physical symptoms at baseline was calculated to be 70% for cases and 23% for controls, which strongly supports the interacting nature of physical symptoms and the development of TMD.

There is a comorbidity between TMD and other chronic pain conditions, with a large overlap between widespread pain/fibromyalgia and TMD pain [35]. More than 50% of individuals in the general population with myofascial jaw pain report concurrent widespread pain [36]. Although we did not address widespread pain or fibromyalgia in the present study, the relationship is relevant since physical symptoms, including those observed in our study, are strongly associated with chronic overlapping pain conditions (COPCs). The term COPCs embraces this overlap and comorbidity between frequently occurring chronic pain conditions that include TMD, irritable bowel syndrome (IBS), fibromyalgia/widespread pain, lower back pain, and headache [37]. Physical symptoms are also more strongly associated with TMD and lower back pain than with fibromyalgia and IBS [38]. In addition to the overlap in physical symptoms, COPCs share psychosocial comorbidities such as higher levels of depression, stress, and anxiety [37]. Even though the cause of the relationship between jaw pain and physical symptoms is still unclear, COPCs could provide a possible explanation worth exploring. Furthermore, awareness of physical symptoms can be of relevance in the assessment of risk factors for TMD in dentistry.

TMD can affect quality of life, and a high prevalence of physical symptoms and psychosocial factors such as depression is seen among TMD patients [34]. Psychosocial variables may also affect the prognosis and are therefore concluded to be important to take into account in the assessment and management of patients [39]. Physical symptoms are also correlated with anxiety and stress, and with depression but to a lesser extent [40]. In our study, depression was not however a predictor for the development or maintenance of jaw pain.

### Gender

Our results regarding female gender being a predictor for jaw pain were expected and are in line with previous results. Women are more likely to report pain in general [30] and to report more physical symptoms both in terms of frequency and intensity [41]. Women have a higher prevalence for acute and chronic TMD [3, 4], and the same pattern is seen for WAD where women experience more chronic WAD symptoms [18]. Pain development is multifactorial, and the reasons for the gender differences in pain development also need to be further explored. Nevertheless, the gender difference in pain development and maintenance is important to take into account in the clinical setting both during history taking, diagnosis, and assessment of prognosis to tailor individual management strategies effectively.

### Strengths and limitations

All individuals who visited the emergency department at Umeå University Hospital, within a well-defined catchment area, with neck pain following a car accident were invited to participate in our study; thus, we regard the study sample probably more representative of the general population than a sample recruited from, for example, a specialist pain unit. Many previous studies on whiplash populations with TMD have included individuals with chronic whiplash. What is unique with the current data is that we follow the individuals with a whiplash trauma from the acute stage, 1 month after trauma, to a possible chronic stage after 2 years. Because this is a trauma group, we had no access to data prior to the trauma and the only option to collect such data would have been to access medical records or ask participants about previous pain which would be affected by recall bias. The controls were recruited from the general population with the only exclusion criteria being previous neck trauma. The mild neck pain intensity [24] and disability among cases and controls could be interpreted as individuals with high neck pain intensity or disability did not want to participate in this study or that the cohort was quite healthy in their neck and did not experience that much neck pain or consequences due to pain. Another possible interpretation is that cases with high neck pain intensity and neck disability were not able to participate since they had other medical issues due to the recent car accident.

## Conclusion

Pain development and maintenance in the jaw region during the period between 1 month and 2 years after whiplash trauma are primarily not related to the trauma itself, but more associated with physical symptoms and general mechanisms behind widespread pain. This emphasizes the importance of a biopsychosocial perspective on pain development. The higher prevalence of jaw pain 1 month after the trauma highlights the importance of early assessment and management to potentially prevent the development of chronic jaw pain after whiplash trauma.

### Supplementary Information

Below is the link to the electronic supplementary material.

Supplementary file1 (DOCX 36 KB)

## Data Availability

The data supporting the findings of this study are available on request from the authors.
