# Supplementary material for: Whiplash trauma did not predict jaw pain after 2 years: an explorative study
Source: Clin Oral Investig. 2024 Feb 22;28(3):165. doi: 10.1007/s00784-024-05555-z (PMC10881702; doi:10.1007/s00784-024-05555-z)
Supplement: Supplementary file 1 — Supplementary file1 (DOCX 36 KB) [file 784_2024_5555_MOESM1_ESM.docx]

STROBE Statement—checklist of items that should be included in reports of observational studies

|  | Item No. | Recommendation | Page  No. | Relevant text from manuscript |
| --- | --- | --- | --- | --- |
| **Title and abstract** | 1 | (*a*) Indicate the study’s design with a commonly used term in the title or the abstract | Title and abstract | Exploring predictive factors: Explorative study |
|  |  | (*b*) Provide in the abstract an informative and balanced summary of what was done and what was found | Abstract | What was done: One hundred nineteen cases (73 women) and 104 controls (59 women), mean age 34.9 years (SD 13.9), attended baseline and two-year follow-up examinations. /…/ Jaw pain was assessed by two validated screening questions answered with “yes” or “no”. A logistic regression analysis was used to predict the outcome variable jaw pain (yes/no) after two years.  What was found: Since a previous whiplash trauma alone did not predict development and maintenance of jaw pain over a two-year period, but non-specific physical symptoms did, the development of jaw pain in connection with a whiplash trauma needs to be seen in a biopsychosocial perspective and early interventions are recommended. |
| Introduction | | | |  |
| Background/rationale | 2 | Explain the scientific background and rationale for the investigation being reported | 3,4 | Approximately 20% of individuals with TMD onset will develop chronic jaw pain, and women have a higher risk of developing chronic jaw pain. /.../ However, currently knowledge is sparse on risk factors for the development of chronic jaw pain./…/  The most frequent WAD symptoms are neck pain, neck disability and headache /…/ however pain can also occur in other body regions including the orofacial area /…/  Most previous studies on the relationship between TMD and WAD have evaluated these conditions separately or in cross-sectional settings/ |
| Objectives | 3 | State specific objectives, including any prespecified hypotheses | 4 | The aim of the present study was to explore predictive factors, including whiplash trauma, for development and maintenance of jaw pain over a two-year period. |
| Methods | | | |  |
| Study design | 4 | Present key elements of study design early in the paper | 4 | Cohort, cases and controls, two- year follow-up |
| Setting | 5 | Describe the setting, locations, and relevant dates, including periods of recruitment, exposure, follow-up, and data collection | 4 | The cases had visited the emergency department at Umeå University hospital, Sweden /…/ The baseline assessment for cases was performed within a month after trauma between December 2010 and January 2016 (controls were recruited parallel to this time frame) and the two-year follow-up was between December 2012 and January 2018. |
| Participants | 6 | (*a*) *Cohort study*—Give the eligibility criteria, and the sources and methods of selection of participants. Describe methods of follow-up  *Case-control study*—Give the eligibility criteria, and the sources and methods of case ascertainment and control selection. Give the rationale for the choice of cases and controls  *Cross-sectional study*—Give the eligibility criteria, and the sources and methods of selection of participants | 4 | The cases had visited the emergency department at Umeå University hospital, Sweden with a whiplash trauma within 72 hours following a car accident. The cases were recruited through the hospital’s Injury Data Base and the controls were recruited from the general population via advertising./…/ Inclusion criteria for both cases and controls were age 18-70 years, living in Umeå municipality and having an understanding of the written and spoken Swedish language. The exclusion criterion was a neck fracture (WAD grade IV) for cases and a previous neck trauma for controls |
|  |  | (*b*) *Cohort study*—For matched studies, give matching criteria and number of exposed and unexposed  *Case-control study*—For matched studies, give matching criteria and the number of controls per case | N/A |  |
| Variables | 7 | Clearly define all outcomes, exposures, predictors, potential confounders, and effect modifiers. Give diagnostic criteria, if applicable | 5, 6 | Jaw pain was assessed by two questions on pain, answered with “yes” or “no”, from the validated screening questions for TMD (3Q/TMD) /…/Factors included in the analyses were group (case vs. control), gender (women vs. men), neck disability (NDI, 0-100), current neck pain intensity (NRS, 0-10), depression (SCL-90-R, 0-4) and physical symptoms (SCL-90-R, 0-4). The model was adjusted for age (years) and education level (elementary school/secondary school vs. university degree). |
| Data sources/ measurement | 8* | For each variable of interest, give sources of data and details of methods of assessment (measurement). Describe comparability of assessment methods if there is more than one group | *5* | Validated questionnaires were used that are recommended in the standardized examination Research Diagnostic Criteria for TMD (RDC/TMD) |
| Bias | 9 | Describe any efforts to address potential sources of bias | 4,5 | Confirmation bias: During the data collection, group allocation (case/control) was blinded and during analysis the participants remained pseudoanonymous. |
| Study size | 10 | Explain how the study size was arrived at | 10 | All individuals who visited the emergency department at Umeå University Hospital with neck pain following a car accident were invited to participate in our study |

Continued on next page

| Quantitative variables | 11 | Explain how quantitative variables were handled in the analyses. If applicable, describe which groupings were chosen and why | 6 |  |
| --- | --- | --- | --- | --- |
| Statistical methods | 12 | (*a*) Describe all statistical methods, including those used to control for confounding | 6 | A logistic regression analysis was used to predict for the outcome variable jaw pain (yes/no) at two years/…/age was modelled using restricted cubic splines with three nodes. |
|  |  | (*b*) Describe any methods used to examine subgroups and interactions | 6 | Interaction terms were included in the analyses  Descriptive statistics was used to characterise the study population |
|  |  | (*c*) Explain how missing data were addressed | 6, Fig. 1 | Individuals with missing data at follow-up were not included in the analyses.  Loss to follow-up are seen in Figure 1 |
|  |  | (*d*) *Cohort study*—If applicable, explain how loss to follow-up was addressed  *Case-control study*—If applicable, explain how matching of cases and controls was addressed  *Cross-sectional study*—If applicable, describe analytical methods taking account of sampling strategy | 6 | Individuals with missing data at follow-up were not included in the analyses. |
|  |  | (*e*) Describe any sensitivity analyses |  |  |
| Results | | | | |
| Participants | 13* | (a) Report numbers of individuals at each stage of study—eg numbers potentially eligible, examined for eligibility, confirmed eligible, included in the study, completing follow-up, and analysed | 4, Figure 1 | The initial cohort consisted of 292 individuals (176 cases and 116 controls. Of these, 223 individuals (132 women and 91 men, that entailed 119 cases (73 women and 46 men, and 104 controls (59 women and 45 men attended the two-year follow-up and were thus included in the analyses |
|  |  | (b) Give reasons for non-participation at each stage | N/A |  |
|  |  | (c) Consider use of a flow diagram | Figure 1 |  |
| Descriptive data | 14* | (a) Give characteristics of study participants (eg demographic, clinical, social) and information on exposures and potential confounders | Table 1, 2, Fig 2 | Age, gender, neck pain and disability, jaw pain baseline and follow-up and psychological variables. |
|  |  | (b) Indicate number of participants with missing data for each variable of interest | N/A |  |
|  |  | (c) *Cohort study*—Summarise follow-up time (eg, average and total amount) | 4 | 2 years |
| Outcome data | 15* | *Cohort study*—Report numbers of outcome events or summary measures over time |  |  |
|  |  | *Case-control study—*Report numbers in each exposure category, or summary measures of exposure | *N/A* |  |
|  |  | *Cross-sectional study—*Report numbers of outcome events or summary measures |  |  |
| Main results | 16 | (*a*) Give unadjusted estimates and, if applicable, confounder-adjusted estimates and their precision (eg, 95% confidence interval). Make clear which confounders were adjusted for and why they were included | Table 3 | 95% CI |
|  |  | (*b*) Report category boundaries when continuous variables were categorized | N/A |  |
|  |  | (*c*) If relevant, consider translating estimates of relative risk into absolute risk for a meaningful time period |  |  |

Continued on next page

| Other analyses | 17 | Report other analyses done—eg analyses of subgroups and interactions, and sensitivity analyses | N/A |  |
| --- | --- | --- | --- | --- |
| Discussion | | | | |
| Key results | 18 | Summarise key results with reference to study objectives | 7 | From this explorative study that evaluated predictive factors for development and maintenance of jaw pain the main finding was that a previous whiplash trauma alone did not predict jaw pain over a two-year period. Physical symptoms and female gender did however increase the risk for jaw pain, regardless of the other evaluated factors. |
| Limitations | 19 | Discuss limitations of the study, taking into account sources of potential bias or imprecision. Discuss both direction and magnitude of any potential bias | 10 |  |
| Interpretation | 20 | Give a cautious overall interpretation of results considering objectives, limitations, multiplicity of analyses, results from similar studies, and other relevant evidence | 7-11 |  |
| Generalisability | 21 | Discuss the generalisability (external validity) of the study results | 10 | All individuals who visited the emergency department at Umeå University Hospital with neck pain following a car accident were invited to participate in our study; thus the study sample is probably more transferrable to the general population than a sample recruited from, for example, a specialist pain unit. |
| Other information | |  | | |
| Funding | 22 | Give the source of funding and the role of the funders for the present study and, if applicable, for the original study on which the present article is based | 11 | The authors disclosed receipt of the following financial support for the research, authorship, and/or publication of this article: Västerbotten County Council, Grant Number: RV-909851 and VLL-324631; Folksam Research Foundation and The Swedish Association for Survivors of Accident and Injury (RTP). |

*Give information separately for cases and controls in case-control studies and, if applicable, for exposed and unexposed groups in cohort and cross-sectional studies.

**Note:** An Explanation and Elaboration article discusses each checklist item and gives methodological background and published examples of transparent reporting. The STROBE checklist is best used in conjunction with this article (freely available on the Web sites of PLoS Medicine at http://www.plosmedicine.org/, Annals of Internal Medicine at http://www.annals.org/, and Epidemiology at http://www.epidem.com/). Information on the STROBE Initiative is available at www.strobe-statement.org.
